# Hepatitis B Reactivation in a US Cohort of People With HIV and Hepatitis B Core Antibody After Switch to Antiretroviral Therapy Without Hepatitis B Activity

**DOI:** 10.1093/cid/ciag001

**Published:** 2026-02-24

**Authors:** Rachel V Denyer, Janet P Tate, Debra A Benator, Joseph K Lim, Amy Weintrob

**Affiliations:** Infectious Diseases Section, Washington VA Medical Center, Washington D.C., USA; School of Medicine and Health Sciences, The George Washington University, Washington D.C., USA; Research Department, VA Connecticut Healthcare System, West Haven, Connecticut, USA; Yale School of Medicine, Yale University, New Haven, Connecticut, USA; Infectious Diseases Section, Washington VA Medical Center, Washington D.C., USA; School of Medicine and Health Sciences, The George Washington University, Washington D.C., USA; Yale School of Medicine, Yale University, New Haven, Connecticut, USA; Infectious Diseases Section, Washington VA Medical Center, Washington D.C., USA; School of Medicine and Health Sciences, The George Washington University, Washington D.C., USA

**Keywords:** HIV, hepatitis, antiretroviral, reactivation, core antibody

## Abstract

**Background:**

One in 3 people with human immunodeficiency virus (HIV-1; PWH) are hepatitis B (HBV) core antibody positive (anti-HBc+) and surface antigen negative (HBsAg−) suggesting prior exposure. HBV reactivation can occur in this group if nucleos(t)ide reverse transcriptase inhibitor antiretrovirals (ARV) active against both HIV and HBV are stopped. We describe HBV reactivation in anti–HBc+/HBsAg− PWH following switch from ARV with HBV activity to ARV without HBV activity.

**Methods:**

We identified an at-risk cohort of 5986 anti-HBc+ participants switched from HBV-active to non–HBV-active ARV on or before 31 December 2023 and HBsAg− on the most recent result preceding switch from 63 153 PWH in the Veterans Aging Cohort Study. We defined HBV reactivation as HBV DNA detection or HBsAg+ result at any time following switch. HBV-active ARV included lamivudine, emtricitabine, or tenofovir.

**Results:**

Forty (0.67%) anti-HBc+/HBsAg− PWH experienced HBV reactivation after switch to non–HBV-active ARV, with median time to reactivation 8.9 months (interquartile range 5.5–26.7). The rate of HBV reactivation was 25.1 per 10 000 person-years (95% confidence interval [CI], 18.4–34.3). Prespecified subgroup analyses revealed higher rates per 10 000 person-years of HBV reactivation in those HBsAg+ in the remote past with no hepatitis B surface antibody positive (anti-HBs+) result (321; 95% CI, 120–855) versus subgroups never previously HBsAg+ or anti-HBs+ (38.0; 95% CI, 22.9–63.0), or anti-HBs+ but never HBsAg+ (17.4; 95% CI, 11.2–27.0).

**Conclusions:**

Overall risk of HBV reactivation appears low after switch from HBV-active to non–HBV-active ARV among anti-HBc+ PWH with no prior HBsAg+. Our results inform provider-patient discussion about HBV reactivation risk when considering ARV switching.

## Teaser

In people with HIV with positive hepatitis B core antibody and negative hepatitis B surface antigen results, hepatitis B reactivation is infrequent following switch from antiretrovirals with hepatitis B activity to those without hepatitis B activity.

## Graphical Abstract

**Graphical Abstract:**
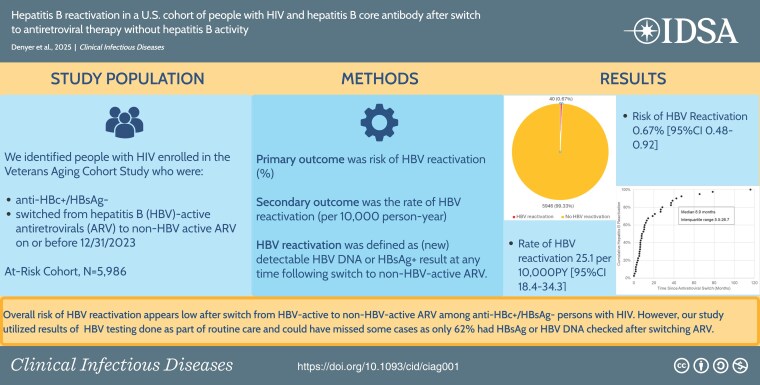
This graphical abstract is also available at Tidbit: https://tidbitapp.io/institutional-portal/clinical-infectious-diseases/tidbits/hepatitis-b-reactivation-in-a-u-s-cohort-of-people-with-hiv-and-hepatitis-b-core-antibody-after-switch-to-antiretroviral-therapy-without-hepatitis-b-activity/update

**(See the Editorial Commentary by Mohareb and Boyd on pages e743–5.)**

Approximately 1 in 3 people with human immunodeficiency virus (HIV-1; PWH) are hepatitis B (HBV) core antibody positive (anti-HBc+) and surface antigen negative (HBsAg−), compared to an estimated 3.5% to 4% for the US population overall [1, 2]. In a large cohort of 5222 PWH from the US Department of Defense, 347 (7%) were isolated anti-HBc+ (HBsAg− and HBV surface antibody negative [anti-HBs–]), and 1073 (21%) had evidence of resolved hepatitis B infection (concurrently anti-HBc+ and HBV surface antibody positive [anti-HBs+]) [2]. There are multiple reports of both HBV reactivation and occult HBV infection (HBsAg− with detectable HBV DNA) in patients with isolated anti-HBc+ status [2, 3, 5–8]. HBV reactivation can occur due to the persistence of covalently closed circular DNA within hepatocytes following acute HBV infection [4]. Occult HBV infection has been reported in 10% to 27% of patients with isolated cAb positivity [3, 7, 9]. Cases of HBV reactivation have occurred spontaneously in PWH with isolated cAb positivity [6], after antiretroviral (ARV) initiation, and in response to cessation of HBV-active ARV regimens [2, 8, 10]. Immune reconstitution after ARV initiation and the frequent use of tenofovir-based ARV, with activity against both HIV and hepatitis B, contributes to higher rates of HBV surface antigen loss of 10% to 54% among PWH, compared to those without HIV [11–13].

Most PWH receive 3-drug ARV including nucleos(t)ide reverse transcriptase inhibitors (NRTIs) such as tenofovir alafenamide (TAF), tenofovir disoproxil fumarate (TDF), lamivudine (3TC), or emtricitabine (FTC), which are dually active against HBV and HIV, though tenofovir is the drug of choice for PWH with HBV co-infection [14]. Previously, ARV that included protease inhibitors in combination with integrase strand transfer inhibitors were used as salvage therapy, but current HIV treatment guidelines allow for broader use of NRTI-sparing ARV including dolutegravir with rilpivirine and long-acting injectable intramuscular cabotegravir with rilpivirine [15]. These regimens may appeal to clinicians and PWH concerned about drug toxicities with long-term use of standard 3-drug regimens and help PWH who have difficulty taking daily oral ARV; however, they lack activity against HBV. Switching anti-HBc+/HBsAg− PWH to NRTI-sparing regimens places them at potential risk for HBV reactivation, but this risk is not well-defined based on data from the SWORD 1, SWORD 1, ATLAS, and FLAIR clinical trials as these trials excluded anti-HBc+/HBsAg−/anti-HBs− participants [16–18].

In a small single-center case series, 3 of 38 anti-HBc+/HBsAg− PWH developed low-level HBV viremia after switching to long-acting cabotegravir-rilpivirine, including one anti-HBs+ PWH with titer of 115 mIU/L [19]. A small prospective study in Cameroon examined PWH switched from NRTI-based 3-drug ARV to ritonavir-boosted darunavir monotherapy and found 10% (6 of 60) of PWH who were anti-HBc+/HBsAg− prior to switch either became HBsAg+ or developed detectable HBV DNA indicative of HBV reactivation [20]. No similar data are available from larger studies to guide clinicians and patients in understanding the risk of HBV reactivation in anti-HBc+/HBsAg− PWH when switching from HBV-active ARV to regimens lacking HBV activity. We aimed to understand the risk of HBV reactivation in anti-HBc+/HBsAg− PWH after switch from HBV-active to non–HBV-active ARV.

## METHODS

### Study Design and Population

This study used data from 63 153 PWH enrolled in the Veterans Aging Cohort Study, a longitudinal virtual electronic health record cohort of US Veterans, which has been described previously [21].

### Inclusion and Exclusion Criteria

We identified 43 616 participants living as of 1 January 2005 who had received ARV from the Veterans Health Administration (VHA), of whom 18 637 had a positive cAb. From this group, we identified a cohort of 5986 PWH at risk for hepatitis B reactivation who met the following inclusion criteria: (1) HBV-active ARV stopped and non–HBV-active ARV started before 31 December 2023 on at least 1 occasion, (2) anti-HBc+ result predates ARV switch, and (3) were HBsAg− (and if checked also had negative/undetectable HBV DNA) on nearest test before the date of ARV switch. We excluded those who were either HBsAg+ or had detectable HBV DNA on the result before ARV switch because these groups would not be expected to switch to ARV lacking HBV activity, but we did not exclude these participants if they had a detectable HBV DNA result or were HBsAg+ in the more remote past as long as they had a more recent HBV DNA or HBsAg result that was negative.

HBV-active ARV included at least 1 of 3TC, FTC, TDF, or TAF. Stopping of HBV-active ARV was determined using an algorithm that identified prescription fill data with a gap in HBV-active ARV of at least 60 days. If no alternative non–HBV-active ARV was prescribed after the HBV-active ARV was stopped, this was considered a treatment interruption and was not counted as an ARV switch eligible for inclusion. Participants with treatment interruptions were not excluded if they also had ARV switches that met the inclusion criteria. All participants with HBV reactivation occurring at any time after the date of switch underwent detailed review of individual ARV fills by study team to confirm ARV regimen before HBV reactivation. We did this to avoid missing cases where the ARV regimen may have been switched back to HBV-active ARV due to clinical suspicion for HBV reactivation before availability of repeat HBV testing results, and to avoid misattributing HBV reactivation to non-HBV-active ARV if the participant was no longer receiving that regimen.

### Outcome Definitions

#### Primary Outcome

The primary outcome was overall risk of HBV reactivation, expressed as the number of persons with HBV reactivation as a percentage of the total number of persons in the at-risk cohort. We defined HBV reactivation as any detectable HBV DNA or HBsAg+ result (either positive qualitative result or any numeric quantitative result above the lower limit of quantification for the assay) at any time following switch to non–HBV-active ARV that occurred while receiving the non–HBV-active regimen.

#### Secondary Outcomes

Secondary outcomes included: (1) detailed description of HBV reactivation including time from ARV switch to HBV reactivation, changes to antivirals/ARV following hepatitis B reactivation, and clinical outcomes (hepatitis flare, hospitalization within 30 days, and 90-day all-cause mortality following HBV reactivation); (2) estimating the rate of HBV reactivation (events per 10 000 person-years) in the at-risk cohort; and (3) prespecified subgroup analysis comparing HBV reactivation risk stratified by prior HBV serology results, hypothesizing that anti-HBc+ PWH with a previous anti-HBs+ result may have lower risk for HBV reactivation relative to those with no anti-HBs+ result. Hepatitis flare was defined as peak ALT elevation to over 100 U/L and at least 3 times’ baseline result based on the definition from the 2018 guidelines from the American Association for the Study of Liver Diseases [22].

### Covariates

We collected baseline data at time of ARV switch, including age, sex, race/ethnicity, CD4 count, and HIV RNA viral load. We included CD4 count and HIV RNA viral load results from 365 days before the ARV switch to 30 days after, with the closest result to date of switch used if multiple results were available.

### Statistical Analysis

The rate of HBV reactivation was calculated as the total time without HBV-active ARV following the date of switch from an HBV-active to a non–HBV-active regimen. Follow-up was censored at the earliest of death, HBV reactivation, 90 days after the most recent prescription for non–HBV-active ARV was filled, or 31 December 2023. When analyzing the timing and rate of HBV reactivation, if participants in the at-risk cohort had multiple eligible ARV switches we used either the closest date of switch to HBV reactivation or a randomly selected eligible switch in participants without HBV reactivation. Clinical outcomes used the VHA Corporate Data Warehouse laboratory results, International Classification of Diseases 9/10 codes at hospital discharge, and recorded dates of death. To evaluate for other known HBV reactivation triggers, we reviewed all prescription medications for the year preceding HBV reactivation to identify hepatitis C treatment with direct acting antivirals or immunosuppressive medications, which were classified into low-, medium-, or high-risk for reactivation according to published guidelines [23]. Confidence intervals for risk/rates of HBV reactivation were calculated using OpenEpi Version 3, all other statistical analysis used SAS version 9.4.

### Ethics

The Veterans Aging Cohort Study was approved by the Yale University and VA Connecticut Healthcare System institutional review boards. Studies were conducted in accordance with the local legislation and institutional requirements. The ethics committee/institutional review board waived the requirement of written informed consent from the participants or the participants’ legal guardians/next of kin because the study used electronic health record data; there was no direct patient contact.

## RESULTS

### Participant Characteristics

**Figure 1.**
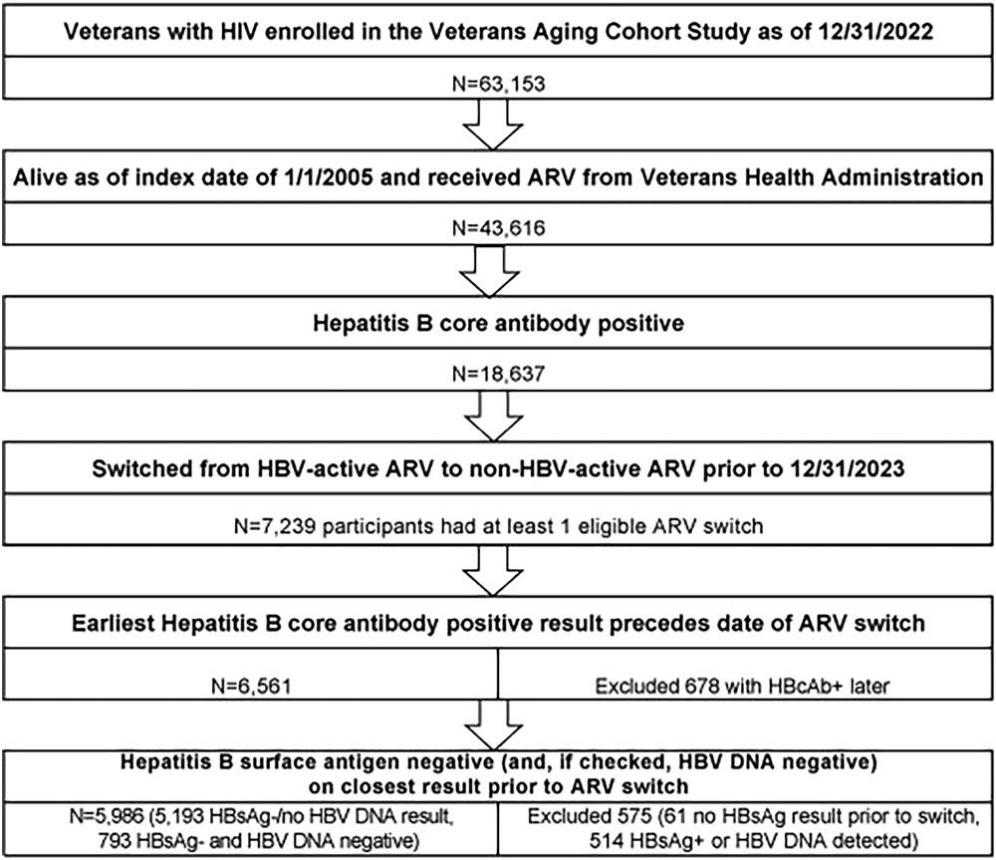
Selection of participants for the at-risk cohort. Abbreviations: anti-HBc+, hepatitis B core antibody positive; ARV, antiretrovirals; HBV, hepatitis B virus; HBV DNA, hepatitis B DNA.

We identified an at-risk cohort of 5986 participants with HIV who were previously anti-HBc+ with no evidence of active hepatitis B and switched from HBV-active ARV to non–HBV-active ARV before 31 December 2023, as outlined in Figure 1. The preswitch regimen included TDF/TAF in 3612 participants (60%) and FTC/3TC with neither TAF nor TDF in 2368 participants (40%). Summary characteristics for this at-risk cohort are presented in Table 1.

**Table 1.** Characteristics of the At-Risk Cohort and Hepatitis B Reactivation Cases

	At-Risk Population, N (%)	Hepatitis B Reactivation Cases Among PWH Switched to ART Without Hepatitis B Activity, N (%)
Age at time of ART switch (y):		
<50	1049 (17.5)	5 (12.5)
50–64	3444 (57.5)	20 (50)
≥65	1493 (24.9)	15 (37.5)
Sex		
Male	5895 (98.5)	39 (97.5)
Race/ethnicity		
Non-Hispanic White	1958 (32.7)	13 (32.5)
Non-Hispanic Black	3117 (52.1)	23 (57.5)
Hispanic	424 (7.1)	2 (5.0)
Other	393 (6.6)	1 (2.5)
Unknown	94 (1.6)	1 (2.5)
CD4 count at time of switch^a^		
<200	920 (15.4)	12 (30.0)
200–499	2399 (40.1)	15 (37.5)
≥500	2458 (41.1)	10 (25.0)
Unknown	209 (3.5)	3 (7.5)
HIV RNA viral load at switch (copies/mL)^a^		
<Lower limit of detection	3802 (63.5)	18 (45.0)
Lower limit of detection-1000	1186 (19.8)	8 (20.0)
1000–10 000	322 (5.4)	5 (12.5)
>10 000	527 (8.8)	8 (20.0)
Unknown	149 (2.5)	1 (2.5)
Total	5986	40

Abbreviations: ART, antiretroviral therapy; HIV, human immunodeficiency virus; PWH, people with human immunodeficiency virus.

^a^Within 365 days before the switch to 30 days after, closest result to date of ART switch used if multiple available.

### Incidence and Rate of HBV Reactivation

**Figure 2.**
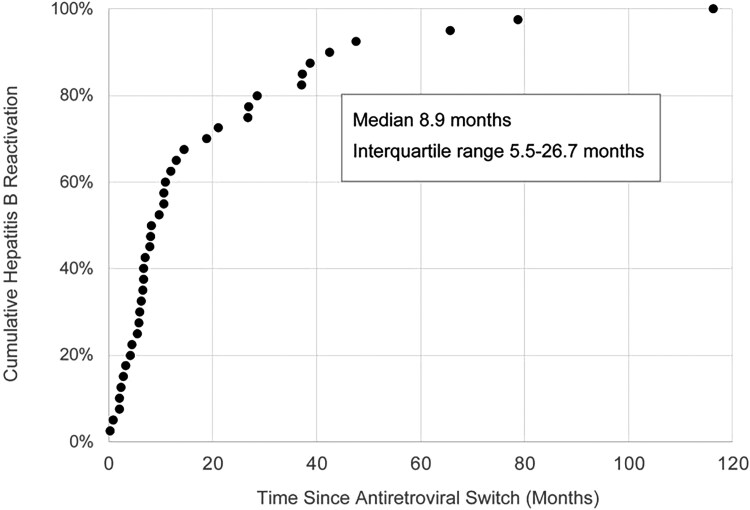
Time elapsed from switch to antiretroviral therapy without hepatitis B activity to hepatitis B reactivation. Each dot represents 1 observed case of hepatitis B reactivation (n = 40).

In the year following ARV switch, at least 1 result for ALT or aspartate aminotransferase was available for 5660 of 5986 participants (95%), with 3840 participants (64%) having 3 or more results available. Repeat HBV DNA or surface antigen results at any time after ARV switch were available for 3718 participants (62%) in the at-risk cohort. Among the 5986, 88 cases of HBV reactivation occurred after a switch to non–HBV-active ARV. In 40 participants (0.67%), this met our primary outcome definition, occurring in participants receiving non–HBV-active ARV at the time of reactivation. Summary characteristics for both the at-risk cohort and these 40 participants experiencing HBV reactivation are presented in Table 1. HBV-active components of the preswitch ARV comprised TDF/TAF alone in 5 participants, 3TC or FTC alone in 18 participants, and TDF/TAF plus either 3TC or FTC in 17 participants experiencing HBV reactivation. The median time to HBV reactivation was 8.9 months after ARV switch (interquartile range 5.5–26.7 months), with most cases of HBV reactivation occurring within 1 year, as shown in Figure 2. An additional 48 cases of HBV reactivation (0.80%) that occurred in the at-risk group among participants no longer receiving non–HBV-active ARV before HBV reactivation were excluded from further analysis: 22 participants were not receiving any ARV (10 of whom had no ARV for over a year before HBV reactivation), 10 participants were receiving 3TC/FTC without tenofovir, 16 participants were receiving TAF/TDF plus 3TC/FTC (of these, 9 had been receiving this for more than a year, 3 participants for 3–12 months, and 4 participants for less than 3 months before the date of HBV reactivation).

We included a total of 15 916 person-years of follow-up of 5938 at-risk participants receiving non–HBV-active ARV after switch. Using this approach, we estimate that the rate of HBV reactivation in our at-risk cohort was 25.1 per 10 000 person years of follow-up (95% CI, 18.4–34.3).

### Clinical Outcomes

Table 2 shows clinical outcomes and subsequent changes to anti(retro)virals amongst those with HBV reactivation. HBV DNA results within 180 days of the date of reactivation were available for 30 of the 40 participants experiencing HBV reactivation and median HBV DNA was 30 796 IU/mL (interquartile range 67–4 149 540). Among 15 of 40 participants with HBV reactivation with ALT elevation to over 100 U/L, median ALT was 538 U/L (range 103–2648). Ten of 40 participants with HBV reactivation (25%) met AASLD criteria for HBV reactivation with hepatitis flare with ALT > 100 IU and 3-fold increase from baseline.

**Table 2.** Clinical Outcomes Among People With HIV Experiencing Hepatitis B Reactivation After Switching to Antiretrovirals Without Hepatitis B Activity

	Hepatitis B Reactivation Cases Among PWH Switched to ART Without Hepatitis B Activity, N (%)
Hepatitis B reactivation criteria met:	
Positive HBsAg alone	14 (35.0)
Positive HBVDNA alone	7 (17.5)
Both HBsAg and HBVDNA detected	19 (47.5)
CD4 count at reactivation^a^	
<200	10 (25.0)
200–499	17 (42.5)
≥500	8 (20.0)
Unknown	5 (12.5)
HIV RNA viral load at reactivation (copies/mL)^a^	
<Lower limit of detection	20 (50.0)
<1000	6 (15.0)
1000–10 000	4 (10.0)
>10 000	9 (22.5)
Unknown	1 (2.5)
Peak HBV DNA (< 180 days after reactivation):	
<1000	14 (35.0)
>1000	1 (2.5)
>10 000	15(37.5)
No data	10 (25.0)
Alanine aminotransferase elevation within 90 days before/after date of reactivation	
ALT > 100	15 (37.5)
ALT >100 and 3-fold increase	10 (25.0)
No data	0 (0.0)
Hospitalization within 30 days before/after date of reactivation^b^	16 (40.0)
All-cause mortality within 90 days before/after date of reactivation	2 (5.0)
Changes to anti(retro)viral treatment after reactivation	
Tenofovir restarted	6 (15.0)
Tenofovir plus 3TC/FTC restarted	15 (37.5)
3TC/FTC alone added (no other hepatitis B active agent)	7 (17.5)
Entecavir added^c^	5 (12.5)
Adefovir added	1 (2.5)
No change	6 (15.0)
Missing data	1 (2.5)
Total	40 (100)

Abbreviations: 3TC, lamivudine; ART, antiretroviral therapy; FTC, emtricitabine; HIV, human immunodeficiency virus; PWH, people with human immunodeficiency virus.

^a^30 days before reactivation to 90 days after, closest result to date of reactivation used if multiple available.

^b^Excluding hospitalizations for mental health diagnoses.

^c^One of the 5 participants started on entecavir also received FTC; the other 4 did not receive any additional hepatitis B active medication.

### Hepatitis B Reactivation Stratified by Prior Hepatitis B Serology Results

Table 3 shows the distribution of participants in the at-risk cohort with and without HBV reactivation stratified by all available HBV serologic testing results ever, prior to the eligible ARV switch. The subgroup with prior HBsAg+ in the remote past and no anti-HBs+ result ever had the highest risk of HBV reactivation at 7.02% with the subgroup with no prior HBsAg+ result and previous anti-HBs+ results being the lowest risk for reactivation at 0.47%. Half of the cases of HBV reactivation (20/40) occurred amongst participants with prior anti-HBs+ who had never had a prior HBsAg+ result, indicating incomplete protection afforded by anti-HBs+ status. Five cases of HBV reactivation (12.5%) occurred in participants who were HBsAg− before ARV switching but had an anti-HBs+ result in the more remote past. We conducted a sensitivity analysis limited to the most recent result for anti-HBs+ and excluding participants with missing anti-HBs data and noted similar trends in rates of reactivation across subgroups (Supplementary Table 1).

**Table 3.** Rate of Hepatitis B Reactivation in People With HIV With Positive Hepatitis B Core Antibody After Switching to Antiretrovirals Lacking Hepatitis B Activity Stratified by Historical Surface Antigen and Surface Antibody Status

Prior Hepatitis B Serology	At-Risk Cohort	Participants With Hepatitis B Reactivation		Follow-Up Time on Non–HBV-Active ARV	Rate of Hepatitis B Reactivation
	N	N	Risk, % [95% CI]^a^	PY	Rate, per 10 000 PY [95% CI]^b^
Never HBsAg+, ever anti-HBs+	4279	20	0.47 [.29–.72]	11 493	17.4 [11.2–27.0]
Never HBsAg+, never anti-HBs+	1467	15	1.02 [.57–1.68]	3952	38.0 [22.9–63.0]
Historical HBsAg+^c^, never anti-HBs+	57	4	7.02 [1.95–17.00]	125	321 [120–855]
Historical HBsAg+, ever anti-HBs+	135	1	0.74 [.02–4.06]	347	28.9 [4.06–205]
Total	5938	40	0.67 [.48–.92]	15 916	25.1 [18.4–34.3]

Abbreviations: anti-HBs+, hepatitis B surface antibody positive; ARV, antiretrovirals; CI, confidence interval; HBsAg+, hepatitis B surface antigen positive; HBV, hepatitis B; HIV, human immunodeficiency virus; PY, person-years.

^a^Fisher exact test.

^b^Rothman/Greenland.

^c^To be eligible, participants had to have negative hepatitis B surface antigen before the switch to antiretroviral regimen without hepatitis B activity, but were not excluded if they had documentation of a prior historical HBsAg+ result in the more remote past.

***Other Factors Associated with Hepatitis B Reactivation.*** One of the 40 participants with hepatitis B reactivation had been taking non–HBV-active ARV for 19 months and hepatitis C treatment with elbasvir and grazoprevir for 4 weeks at the time of reactivation. Eight of 40 participants with HBV reactivation received immunosuppressive medications in the preceding year. Five received immune suppression with low risk for HBV reactivation (4 received low-dose corticosteroids for less than 4 weeks’ duration, 1 received low-corticosteroids for more than 4 weeks with mycophenolate mofetil and tacrolimus). Three received moderate-risk immunosuppression; relative to the date of HBV reactivation, 1 had been on non-HBV-active ARV for 9 months and received moderate-intensity corticosteroids for 3 months prior, 1 had been on non–HBV-active ARV for 10 months and received doxorubicin 9 to 11 months prior, and 1 had been on non–HBV-active ARV for 26 months and received doxorubicin 4 to 6 months prior.

## DISCUSSION

Our study found that 0.67% of anti-HBc+/HBsAg− PWH who switched from HBV-active to non–HBV-active ARV developed HBV reactivation while receiving non–HBV-active ARV. PWH with no prior anti-HBs+ result and prior HBsAg+ results in the remote past (in spite of being HBsAg− before switching to non–HBV-active ARV) had significantly higher risk for HBV reactivation compared to those subgroups with no prior HBsAg+ result ever, though it should be noted that we had less follow-up time in the prior HBsAg+ subgroups due to smaller numbers of participants. Nonetheless, this demonstrates the importance of carefully reviewing all previous HBV serology results and medical records to look for any evidence of active HBV in the past, before cessation of HBV-active ARV. Twenty-one of the 40 participants with HBV reactivation from our at risk cohort had a prior anti-HBs+ result, indicating incomplete immunologic protection from hepatitis B surface antibody. This has been described previously with 4 of 6 PWH experiencing HBV reactivation in Abdullahi et al noted to be anti-HBs+ as well as anti-HBc+/HBsAg− at the time of ARV switch. Additionally, a study of incident HBV infections among 5785 PWH in Texas identified 3 cases of HBV reactivation in previously anti-HBc+/HBsAg− PWH receiving non–HBV-active ARV, of whom 2 had prior anti-HBs+ titers of 18.3 and 58.3 mIU/mL [24]. Vasishta et al also describe HBV reactivation in an anti-HBc+/HBsAg−/anti-HBs+ PWH (previously HBV-vaccinated) 5 months after an ARV switch involving cessation of TDF and FTC for worsening renal function with advanced uncontrolled HIV [10].

We found an incidence rate of 25.1 per 10 000 person-years for HBV reactivation after switching to non–HBV-active ARV, much lower than the 10% incidence described by Abdullahi et al (1090 per 10 000 person-years) [20]. This may reflect Cameroon being a higher endemicity setting for HBV compared to our US veteran population, as well as key methodological differences between the studies. Our study had nearly 100 times the number of participants in our at-risk cohort compared to that of Abdullahi et al, with robust longitudinal laboratory and clinical data from VHA corporate data warehouse, but we were unable to capture HBV testing performed outside of VHA or HBV vaccination records from the US Department of Defense. Although our study lacked control over the timing and frequency of hepatitis B testing performed for routine clinical care which may have limited our ability to detect HBV reactivation, Abdullahi et al collected blood samples prospectively to measure HBV surface antigen and HBV DNA at 4, 12, 24, 36, and 48 weeks postswitch. They found evidence of HBV reactivation in samples collected from 12 to 48 weeks after switch, consistent with our median time to reactivation of 269.5 days.

This study has several limitations because of the methodology and data available through the Veterans Aging Cohort Study. First, our study is observational and HBV reactivation is complex and multifactorial, making it difficult to attribute a causal link between ARV switch and HBV reactivation. However, we did describe possible alternative triggers for the HBV reactivation observed, including hepatitis C treatment, immunosuppression, and ARV cessation (which could lead to HBV reactivation from untreated advanced HIV). Second, we cannot fully exclude the unlikely possibility of a false-positive isolated cAb and subsequent acute hepatitis B infection de novo after switch to non–HBV-active ARV. Third, the use of prescription fill data to determine ARV discontinuation dates as participants could receive medications from pharmacies outside of the VHA system. Fourth, we defined ARV regimen stopping as a lapse of at least 60 days in refilling a given regimen (to avoid mislabeling early/late medication refills of the same ARV as stopping and restarting that regimen) and therefore we may miss brief ARV switches if the prior regimen is restarted less than 60 days later. Fifth, our at-risk cohort included only a small proportion of female participants (1.5%), which may limit generalizability of the results for female PWH. Sixth, although we examined the relationship between prior anti-HBs+ results and HBV reactivation risk in our cohort, we were unable to examine the relationship between HBV vaccination and reactivation directly. Current HIV guidelines recommend all anti-HBc+/HBsAg− PWH who lack immunity should be offered HBV vaccination [14].

Overall, our data inform discussions between providers and anti-HBc+/HBsAg− PWH as to the risk of HBV reactivation when switching to non–HBV-active ARV. Additional research is needed to better understand the timing, incidence, and predictors of HBV reactivation in the context of switching from HBV-active to non–HBV-active ARV in anti-HBc+/HBsAg− PWH, and the role of vaccination in preventing HBV reactivation. Furthermore, prospective studies are needed to identify evidence-based strategies for surveillance or early detection of HBV reactivation via established or novel biomarkers.

## Supplementary Material

ciag001_Supplementary_Data

## Supplementary Data

Supplementary materials are available at *Clinical Infectious Diseases* online. Consisting of data provided by the authors to benefit the reader, the posted materials are not copyedited and are the sole responsibility of the authors, so questions or comments should be addressed to the corresponding author.

## References

[1] Spradling PR, Xing J, Harris AM, Ly KN. Estimated prevalence and number of persons with isolated antibody to hepatitis B core antigen and associated occult hepatitis B, United States, 2001–2018. J Infect Dis 2022; 225:465–9. 34252183 10.1093/infdis/jiab366

[2] Landrum ML, Roediger MP, Fieberg AM, et al Development of chronic hepatitis B virus infection in hepatitis B surface antigen negative HIV/HBV co-infected adults: a rare opportunistic illness. J Med Virol 2011; 83:1537–43. 21739443 10.1002/jmv.22155

[3] Mitsumoto-Kaseida F, Murata M, Takayama K, et al Prevalence and characteristics of occult hepatitis B virus infection in Japanese human immunodeficiency virus-infected patients. J Infect Chemother 2020; 26:28–32. 31279522 10.1016/j.jiac.2019.06.003

[4] Myint A, Tong MJ, Beaven SW. Reactivation of hepatitis B virus: a review of clinical guidelines. Clin Liver Dis (Hoboken) 2020; 15:162–7. 32395244 10.1002/cld.883 PMC7206320

[5] Chamorro AJ, Casado JL, Bellido D, Moreno S. Reactivation of hepatitis B in an HIV-infected patient with antibodies against hepatitis B core antigen as the only serological marker. Eur J Clin Microbiol Infect Dis 2005; 24:492–4. 15990987 10.1007/s10096-005-1355-1

[6] Pei R, Grund S, Verheyen J, Esser S, Chen X, Lu M. Spontaneous reactivation of hepatitis B virus replication in an HIV coinfected patient with isolated anti-hepatitis B core antibodies. Virol J 2014; 11:9. 24444423 10.1186/1743-422X-11-9 PMC3902409

[7] Azadmanesh K, Mohraz M, Aghakhani A, et al Occult hepatitis B virus infection in HIV-infected patients with isolated hepatitis B core antibody. Intervirology 2008; 51:270–4. 18841029 10.1159/000160217

[8] Zaltron S, Cambianica A, Di Gregorio M, et al Case report: an occult hepatitis B virus infection reactivation in an HIV/HCV coinfected patient during an immune reconstitution inflammatory syndrome. Front Cell Infect Microbiol 2023; 13:1143346. 37124041 10.3389/fcimb.2023.1143346 PMC10145166

[9] Lo Re V III, Frank I, Gross R, et al Prevalence, risk factors, and outcomes for occult hepatitis B virus infection among HIV-infected patients. J Acquir Immune Defic Syndr 2007; 44:315–20. 17159655 10.1097/QAI.0b013e31802ea499

[10] Vasishta S, Dieterich D, Mullen M, Aberg J. Brief report: hepatitis B infection or reactivation after switch to 2-drug antiretroviral therapy: a case series, literature review, and management discussion. J Acquir Immune Defic Syndr 2023; 94:160–4. 37345994 10.1097/QAI.0000000000003239

[11] Jain MK, Vigil KJ, Parisot P, et al Incidence and predictors of hepatitis B surface antigen clearance in HIV patients: a retrospective multisite study. Open Forum Infect Dis 2021; 8:ofab116. 34337091 10.1093/ofid/ofab116 PMC8320286

[12] Msomi N, Naidoo K, Yende-Zuma N, et al High incidence and persistence of hepatitis B virus infection in individuals receiving HIV care in KwaZulu-Natal, South Africa. BMC Infect Dis 2020; 20:847. 33198649 10.1186/s12879-020-05575-6 PMC7670610

[13] Wyles DL . Antiretroviral effects on HBV/HIV co-infection and the natural history of liver disease. Clin Liver Dis 2019; 23:473–86. 31266621 10.1016/j.cld.2019.04.004

[14] Panel on Guidelines for the Prevention and Treatment of Opportunistic Infections in Adults and Adolescents with HIV . Guidelines for the Prevention and Treatment of Opportunistic Infections in Adults and Adolescents with HIV. National Institutes of Health, Centers for Disease Control and Prevention, HIV Medicine Association, and Infectious Diseases Society of America. Year 2024. Available at: https://clinicalinfo.hiv.gov/en/guidelines/adult-and-adolescent-opportunistic-infection. Accessed 3 July 2025.

[15] Panel on Guidelines for the Use of Antiretroviral Agents in Adults and Adolescents with HIV . Considerations for Antiretroviral Use in Special Populations. National Institutes of Health, Centers for Disease Control and Prevention, HIV Medicine Association, and Infectious Diseases Society of America. Year 2024. Available at: https://clinicalinfo.hiv.gov/en/guidelines/adult-and-adolescent-opportunistic-infection. Accessed 3 July 2025.

[16] Aboud M, Orkin C, Podzamczer D, et al Efficacy and safety of dolutegravir-rilpivirine for maintenance of virological suppression in adults with HIV-1: 100-week data from the randomised, open-label, phase 3 SWORD-1 and SWORD-2 studies. Lancet HIV 2019; 6:e576–87. 31307948 10.1016/S2352-3018(19)30149-3

[17] Swindells S, Andrade-Villanueva JF, Richmond GJ, et al Long-acting cabotegravir and rilpivirine for maintenance of HIV-1 suppression. N Engl J Med 2020; 382:1112–23. 32130809 10.1056/NEJMoa1904398

[18] Orkin C, Arasteh K, Górgolas Hernández-Mora M, et al Long-acting cabotegravir and rilpivirine after oral induction for HIV-1 infection. N Engl J Med 2020; 382:1124–35. 32130806 10.1056/NEJMoa1909512

[19] Welford E, Yin J, Hill L, Wooten D. 1583. A case series of low-level HBV viremia after switching to long-acting injectable cabotegravir/rilpivirine in patients with HIV, hepatitis B core antibody positivity, and hepatitis B surface antigen negativity. Open Forum Infect Dis 2022; 9:ofac492.106.

[20] Abdullahi A, Fopoussi OM, Torimiro J, Atkins M, Kouanfack C, Geretti AM. Hepatitis B virus (HBV) infection and re-activation during nucleos(t)ide reverse transcriptase inhibitor-sparing antiretroviral therapy in a high-HBV endemicity setting. Open Forum Infect Dis 2018; 5:ofy251. 30377627 10.1093/ofid/ofy251 PMC6201150

[21] Fultz SL, Skanderson M, Mole LA, et al Development and verification of a “virtual” cohort using the National VA Health Information System. Med Care 2006; 44:S25–30. 16849965 10.1097/01.mlr.0000223670.00890.74

[22] Terrault NA, Lok ASF, McMahon BJ, et al Update on prevention, diagnosis, and treatment of chronic hepatitis B: AASLD 2018 hepatitis B guidance. Hepatology 2018; 67:1560–99. 29405329 10.1002/hep.29800 PMC5975958

[23] Reddy KR, Beavers KL, Hammond SP, Lim JK, Falck-Ytter YT; American Gastroenterological Association Institute. American Gastroenterological Association Institute guideline on the prevention and treatment of hepatitis B virus reactivation during immunosuppressive drug therapy. Gastroenterology 2015; 148:215–9. 25447850 10.1053/j.gastro.2014.10.039

[24] Sladic JM, Taylor BS, Thamer M, et al Who is at risk for new hepatitis B infections among people with HIV? Open Forum Infect Dis 2023; 10:ofad375. 37539064 10.1093/ofid/ofad375 PMC10394987

