# Supplementary material for: Hepatitis B Reactivation in a US Cohort of People With HIV and Hepatitis B Core Antibody After Switch to Antiretroviral Therapy Without Hepatitis B Activity
Source: Clin Infect Dis. 2026 Feb 24;82(4):e735–42. doi: 10.1093/cid/ciag001 (PMC13131950; doi:10.1093/cid/ciag001)
Supplement: ciag001_Supplementary_Data [file ciag001_supplementary_data.docx]

**Supplemental Table 1. Rate of Hepatitis B Reactivation in People with HIV with Positive Hepatitis B Core Antibody after Switching to Antiretrovirals Lacking Hepatitis B Activity Stratified by Historical Surface Antigen and Most Recent Surface Antibody.**

| Prior Hepatitis B Serology* | At-Risk Cohort | Participants with Hepatitis B Reactivation | | Follow Up Time on Non-HBV-Active ARV | Rate of Hepatitis B Reactivation |
| --- | --- | --- | --- | --- | --- |
|  | N | N | Risk, % [95% CI]^^^ | PY | Rate, per 10,000 PY [95% CI]^#^ |
| Never HBsAg+, anti-HBs+ on most recent pre-switch result | 3,815 | 16 | 0.4 [0.2-0.7] | 10,235 | 15.6 [9.6-25.5] |
| Never HBsAg+, anti-HBs- or indeterminate on most recent pre-switch result | 1,807 | 18 | 1.0 [0.6-1.6] | 4,848 | 37.1 [23.4-58.9] |
| Historical HBsAg+, anti-HBs- or indeterminate on most recent pre-switch result | 75 | 4 | 5.3 [1.5-13.1] | 160 | 250 [93.8-666] |
| Historical HBsAg+, anti-HBs+ on most recent pre-switch result | 114 | 1 | 0.9 [0.0-4.8] | 304 | 32.9 [4.6-234] |
| Total | 5,811^†^ | 39^†^ | 0.7 [0.5-0.9] | 15,546 | 25.1 [18.3-34.3] |

HBsAg+= Hepatitis B Surface Antigen Positive, anti-HBs+= Hepatitis B Surface Antibody Positive, anti-HBs*-*= Hepatitis B Surface Antibody Negative, HBV=Hepatitis B, ARV= Antiretrovirals, PY= Person Years, CI= Confidence Interval.

*To be eligible participants had to have negative hepatitis B surface antigen prior to switch to antiretroviral regimen without hepatitis B activity, but were not excluded if they had documentation of a prior historical HBsAg+ result in the more remote past.

^^^Fisher’s Exact Test

^#^Rothman/Greenland

^†^Excludes 127 at-risk cohort participants and 1 participant with hepatitis B reactivation who had missing hepatitis B surface antibody results
